# Identifying inflammatory phenotypes associated with lung involvement in systemic sclerosis: k-means clustering approach

**DOI:** 10.3389/fimmu.2025.1568683

**Published:** 2025-05-08

**Authors:** Laura Cano-García, Aimara García-Studer, Sara Manrique-Arija, Fernando Ortiz-Marquez, Rocío Redondo-Rodríguez, Paula Borregón-Garrido, Natalia Mena-Vázquez, Antonio Fernández-Nebro

**Affiliations:** ^1^ Instituto de Investigación Biomédica de Málaga (IBIMA)-Plataforma Bionand, Málaga, Spain; ^2^ UGC de Reumatología, Hospital Regional Universitario de Málaga, Málaga, Spain; ^3^ Departamento de Medicina, Universidad de Málaga, Málaga, Spain

**Keywords:** systemic sclerosis, interstitial lung disease, inflammation, C-reactive protein, hematological indices, inflammatory biomarkers, principal component analysis (PCA), inflammatory phenotypes

## Abstract

**Objective:**

to assess the prognostic impact of clusters of hematologic and biochemical indices on interstitial lung disease (ILD) and respiratory damage associated with systemic sclerosis (SSc)

**Methods:**

Design: We conducted a cross-sectional, uncontrolled study. Participants and Settings: a cohort of patients with SSc (2013 ACR/EULAR criteria) were enrolled in the rheumatology unit of a tertiary hospital in southern Spain. Primary and secondary outcome measures: The primary outcomes were the presence of SSc-ILD and respiratory damage, assessed via the Scleroderma Clinical Trials Consortium Damage Index (SCTC-DI). Inflammatory biomarkers, including both CRP and hematological indices, were obtained. Patients were grouped based on inflammatory phenotypes derived from longitudinal CRP averages and through principal component analysis (PCA) with K-means clustering of cross-sectional variables. Multivariate models were constructed to identify factors associated with SSc-ILD and respiratory damage.

**Results:**

Among 83 patients with SSc, 33.7% had ILD, 30.1% had respiratory damage, and 28.9% were receiving immunosuppressive therapy. A persistent inflammatory phenotype during follow-up was associated with non-Caucasian ethnicity (OR 14.0) and SSc-ILD (OR 17.9). Cross-sectional inflammatory clusters were linked to SSc-ILD (OR 12.8) and damage measured by SCTC-DI (OR 1.2). PC-2, derived from CRP-based variables, was a better predictor of SSc-ILD (OR 3.0) than PC-1, which was based on hematological indices (OR 0.5, non-significant), especially in the presence of anti-Scl70+ antibodies (OR 19.1) and immunosuppressants (OR 42.2). The only variables associated with respiratory damage were average CRP during follow-up (OR 1.2), anti-Scl70+ (OR 7.7), and glucocorticoids (OR 0.2).

**Conclusion:**

CRP-based variables seem to be better predictors of SSc-ILD and respiratory damage than hematological indices.

## 1 Introduction

Systemic sclerosis (SSc) is an uncommon, heterogeneous immune-mediated inflammatory disease (IMID) characterized by co-occurrence of autoimmunity, vasculopathy, and generalized fibrosis. This combination of conditions leads to significant and varied morbidity affecting multiple organ systems, including the skin, musculoskeletal system, digestive system, respiratory system, cardiovascular system, and renal system, and is accompanied by an increased risk of death (1). Involvement of the respiratory and cardiovascular systems contributes most to SSc-related mortality (2). While vasculopathy is an important initial stage of the pathogenesis of SSc, it remains unclear how it is related to inflammation and fibrosis and, therefore, to disease activity and damage (3). It is clearly a priority to identify predictors that identify patients at greater risk of death.

As with IMID in general, the immune response in SSc causes tissues to become inflamed, leading to irreversible damage. However, in contrast with other IMIDs, inflammatory phenomena are clinically less evident in most SSc patients, with a large part of the damage mediated by other factors, such as tissue fibrosis and ischemia, although there is also an association between these factors and inflammation (3).

Clinically useful biomarkers of the activity of SSc similar to those used in other IMIDs have not been identified to date. C-reactive protein (CRP) and other inflammatory markers are limited in their usefulness because they are elevated in a minority of patients. Moreover, they seem to be associated with disease activity in very few organs. In fact, after failing to find differences in acute phase reactant levels between patients with SSc and controls, some authors have speculated that the acute phase response may be impaired in this disease (4). Despite these limitations, some studies found that persistently high CRP levels were associated with a higher risk of early death due to SSc-associated interstitial lung disease (SSc-ILD), independently of demographics, disease duration, cutaneous subtype, treatment, and other factors associated with severity (5). SSc-ILD is more common in non-Caucasian patients and persons with diffuse SSc and is associated with mortality of 40% at 10 years (6). However, a possible association between CRP and respiratory damage formally measured has not been investigated.

Data on the impact of elevated serum CRP levels on severity, activity, and damage in the main organ systems affected in SSc are limited and, sometimes, contradictory. Consistent with some studies, elevated serum CRP could be associated with diffuse SSc in cases of increased vascular resistance, ultrasound-confirmed arthritis, and greater all-cause mortality (7–9).

In addition to CRP and other plasma acute phase reactants, a growing group of highly accessible and inexpensive biomarkers of inflammation based on peripheral blood counts could now provide information on the systemic inflammatory burden in SSc. While these biomarkers have received little attention, they could be useful in specific complications of SSc, monitoring of disease activity, and evaluation of the effects of treatment (10). The inflammatory markers analyzed in this study include various combinations of neutrophils, monocytes, and platelets relative to lymphocytes or hemoglobin. These ratios reflect systemic inflammation and immune activation, with platelets playing a key role beyond hemostasis by releasing pro-inflammatory cytokines (e.g., platelet factor-4) and growth factors such as TGF-β and PDGF, which promote fibroblast activation and extracellular matrix remodeling. Additionally, platelet-neutrophil and platelet-monocyte interactions contribute to endothelial dysfunction and fibrosis by amplifying the inflammatory response. These processes complement CRP as markers of systemic inflammation and collectively sustain the cycle of vascular damage, autoimmunity, and fibrosis in SSc (11). Published data show that the results of blood tests based on these markers could be associated with digital ulcers, SSc-ILD, a more severe clinical course, and greater mortality (12–14).

Finally, the comparative usefulness of CRP and cell counts as markers of inflammation, morbidity, and mortality associated with SSc has received little attention in clinical practice. Therefore, priority should be given to applying these indicators of activity or damage to specific systems caused by the disease.

We hypothesized that in daily clinical practice, serum CRP could better predict SSc-ILD and damage in the respiratory domain than inflammatory indices based on blood counts.

Therefore, the objective of the present study was to assess the prognostic impact of clusters of hematologic and biochemical indices on ILD and respiratory damage associated with SSc. Such an approach would provide the basis for more effective interventions to improve quality of life and reduce mortality.

## 2 Patients and methods

### 2.1 Study design, data source, and sample

We performed a single-center uncontrolled cross-sectional observational study of a cohort of patients with SSC from the Systemic Autoimmune Diseases Unit of the Rheumatology Department of Hospital Regional Universitario de Málaga (HRUM). All the patients signed the informed consent document before being included. The study met the ethical criteria of the Declaration of Helsinki and was approved by the Clinical Research Ethics Committee of HRUM (Code 0343-N-22).

### 2.2 Patient selection

All participating patients were recruited consecutively between March 2022 and May 2024. Patients with SSc were selected based on the 2013 ACR/EULAR classification criteria (score ≥9) (15) and were aged ≥16 years at the onset of their disease. The patients were stratified as having limited cutaneous systemic sclerosis (lcSSc) or diffuse cutaneous systemic sclerosis (dcSSc) according the 2001 LeRoy and Medsger classification criteria (16, 17). Patients with inflammatory diseases other than SSc were excluded, although secondary Sjögren syndrome associated with SSc were allowed. Patients with active infection or pregnancy were also excluded.

### 2.3 Study protocol

At HRUM, patients with SSc are generally seen according to a pre-established protocol for collection of clinical, anthropometric, and analytical data every 3 to 6 months or more frequently if clinically necessary. The most complex patients are usually evaluated or treated by several disciplines through review boards for ILD, pulmonary artery hypertension, or nephritis.

The referring nurse or rheumatologist invited the patients to participate in the study. Clinical data were collected once the selection criteria were confirmed. A specific data collection protocol was used for this study. At the nursing clinic, questionnaires were completed, anthropometric parameters were measured, and biological samples were taken after a fast of ≥8 hours.

### 2.4 Patient and public involvement

Patients or the public were not involved in the design, or conduct, or reporting, or dissemination plans of our research.

### 2.5 Main outcome measure and covariates

#### 2.5.1 Main outcome variables

The primary outcome measures were the presence of SSc-ILD and cumulative respiratory damage.

The presence of ILD and specific lung patterns were defined according to the revised criteria of the American Thoracic Society/European Respiratory Society International Multidisciplinary Consensus Classification of the Idiopathic Interstitial Pneumonias, based on lung biopsy or high-resolution computed tomography (HRCT) (18). The 3 specific patterns were nonspecific interstitial pneumonia (NSIP), usual interstitial pneumonia (UIP), and other patterns, such as bronchiolitis obliterans, organizing pneumonia, lymphocytic interstitial pneumonia, and mixed patterns. The pulmonary function tests (PFTs) included full spirometry, expressed as percent predicted and adjusted for age, sex, and height. Forced vital capacity (FVC) was considered abnormal when <80% predicted. The diffusing capacity of the lungs for carbon monoxide was evaluated using the single-breath method (DLCO-SB) and considered abnormal when <80% predicted (19).

Cumulative respiratory damage was defined according to the Scleroderma Clinical Trials Consortium Damage Index (SCTC-DI), which includes the following as respiratory damage: moderate-severe ILD with an extension of >20% in the chest HRCT with or without FVC <70% (not due to respiratory muscle weakness) for more than 6 months or need for home oxygen (20).

The secondary outcome measures were CRP-related biomarkers of inflammation and blood counts. Inflammation was assessed based on CRP values (normal value < 5 mg/L) at the cut-off and at each of the previous visits during follow-up. Serum CRP values at the cut-off were also adjusted for serum levels of albumin and prealbumin as the ratios of CRP to albumin and CRP to prealbumin. Similarly, we collected automated cell counts for neutrophils, monocytes, and platelets in peripheral blood. These were used to calculate the following: neutrophil-to-lymphocyte ratio (NLR), platelet-to-lymphocyte ratio (PLR), monocyte-to-lymphocyte ratio (MLR), platelet-to-hemoglobin ratio (PHR), systemic immune-inflammation index (SII), and pan-immune-inflammation value (PIV). The NLR was calculated as the neutrophil count (10^9^/mL)/lymphocyte count (10^9^/ml). The MLR was calculated as the monocyte count (10^9^/mL)/lymphocyte count (10^9^/mL). The PLR was calculated as the platelet count (10^9^/mL)/lymphocyte count (10^9^/mL). The PHR was calculated as the platelet count (10^9^/mL)/hemoglobin concentration in g/L (PHR). The SII was calculated as [neutrophil count (10^9^/mL) × platelet count (10^9^/mL)]/lymphocyte count (10^9^/mL). Finally, the PIV was calculated based on the following equation: [neutrophil count (10^9^/mL) × platelet count (10^9^/mL) × monocyte count (10^9^/mL)]/lymphocyte count (10^9^/mL). Anemia was diagnosed based on reduced hemoglobin concentrations (<12.0 g/dL for females and <13.0 g/dL for males).

Depending on the degree of inflammation according to the cross-sectional or longitudinal variables, patients were classified in 2 ways: (1) In patients with ≥3 consecutive serum CRP values at the visits before the cut-off, longitudinal CRP values were summarized as an average, and patients were classified into 3 inflammatory phenotypes according to the percentage of visits with abnormal serum CRP values, a previous EUSTAR study (5). A patient with SSc was classified as having a persistent inflammatory phenotype if the CRP values were ≥5 mg/L at ≥80% of the visits, an intermediate inflammatory phenotype if CRP was ≥5 mg/L at 20-80% of the visits, and a noninflammatory phenotype if the CRP was ≥5 mg/L at <20% of the visits (5). Additionally, CRP-derived indices were calculated by adjusting CRP values for albumin and prealbumin but only point-in-time CRP values were corrected. We also classified patients into 2 groups, inflammatory and noninflammatory, depending on the values obtained at the cut-off for the hematological indices and serum CRP. This classification was based on a principal components analysis (PCA) followed by a k-means test.

### 2.6 Sociodemographic and comorbidity variables

Sociodemographic data were carefully collected from all participants and included age, sex, race/ethnicity, educational level, smoking/drinking, and income. We recorded general comorbid conditions using the age-adjusted Charlson comorbidity index (age-CCI), which takes into account 19 predefined clinical conditions with differently weighted values adapted according to specific age ranges (21, 22). An age-adjusted CCI >2 has been associated with a lower 10-year survival rate, which decreases proportionally with an increasing age-adjusted CCI value (i.e., 1, 2, 3, 4, 5, 6, and ≥7 are associated with survival rates of 96%, 90%, 77%, 53%, 21%, 2%, and 0%, respectively). In addition, we considered traditional cardiovascular risk factors, such as smoking, obesity, arterial hypertension, diabetes mellitus, dyslipidemia, and sedentary lifestyle (23). Cardiovascular risk was evaluated using the European risk assessment score (SCORE) (24).

#### 2.6.1 Other variables associated with SSc

SSc-related variables included data on disease duration counting from the first non-Raynaud symptom, clinical manifestations, laboratory test results, and specific treatments during follow-up. The degree of skin fibrosis was measured using the modified Rodnan skin score (MRSS) (25). We also recorded the presence or absence of the main clinical manifestations occurring up to the study visit, including PFTs at the cut-off, namely, FVC, forced expiratory volume in the first second of expiration (FEV_1_), diffusing capacity of the lungs for carbon monoxide (DLCO), alone and with the carbon monoxide transfer coefficient (KCO). Oxygen saturation was determined using pulse oxymetry. Direct determination of pulmonary artery systolic pressure (PASP) was based on transthoracic echocardiography during the previous 6 months in patients with tricuspid valve regurgitation. PASP values were considered normal if 18 to 25 mmHg, slightly elevated if 35 to 40 mmHg, moderately elevated if they were 40 to 60 mmHg, and severe if >60 mmHg (26).

Disease-induced damage was measured using the SCTC-DI. This index comprises 23 items with different weights in 6 organ systems (musculoskeletal/skin, vascular, gastrointestinal, respiratory, cardiovascular, and renal). Low, medium, and high SCTC-DI scores are defined as <5, 6-12, and ≥13, respectively, with a maximum score of 55 (20).

We also recorded values for antinuclear antibodies (ANA) and specific autoantibodies, namely, anti-U1RNP, anti-anticentromere, anti-Scl70, anti-tRNA polymerase III, anti-PM-Scl, Anti-Ro52, anti-Ro60, anti-La, and anti-aminoacyl-tRNA synthetase. All treatments received since disease onset were recorded, including, but not limited to, vasodilators, endothelin receptor antagonists, antifibrotic drugs, corticosteroids, methotrexate, and other biologic and synthetic immunosuppressants. The treatments received since initiation of follow-up were classified in different ways: (1) all immunosuppressants: methotrexate, cyclophosphamide, mycophenolate mofetil, azathioprine, rituximab, calcineurin inhibitors, and tocilizumab. (2) immunosuppressants other than methotrexate: cyclophosphamide, mycophenolate mofetil, azathioprine, rituximab, calcineurin inhibitors, and tocilizumab.

### 2.7 Statistical analysis

A comprehensive descriptive analysis was conducted for the main variables. The frequencies of the qualitative variables are reported as the number of observations and percentages. Quantitative variables are presented as mean (standard deviation [SD]) or median (interquartile range [IQR]). The normal distribution of the data was confirmed using the Kolmogorov-Smirnov test. Correlations between the quantitative variables were assessed using Spearman’s rho. Subgroups of qualitative variables were compared using the Pearson χ^2^ test (or Fisher’s exact test when applicable), and continuous quantitative variables were assessed using the Mann-Whitney test (with Yates’ correction, if appropriate), or the *t* test for independent samples, as applicable. The Kruskal-Wallis test or 1-way analysis of variance (ANOVA) was used, as needed, to calculate the mean score for the persistent inflammatory phenotypes against different variables. Scheffé’s method or the Games-Howell *post hoc* test was applied for multiple pairwise comparison on the significant ANOVA data sets, depending on whether or not the assumption of homoscedasticity was met. Multiple pairwise comparisons in the cross tables for the 3 inflammatory phenotypes were performed using a Bonferroni-adjusted p value.

Given the high number of and collinearity between inflammation-related variables, a dimensional reduction was performed using principal component analysis (PCA) with the 9 inflammatory markers obtained at the cut-off by grouping them into 2 blocks: cell indices and serum CRP variants. The whole blood counts were excluded from the PCA because they were used in the calculation of the hematological ratios. We also excluded average CRP from the model because it was a longitudinal variable obtained during the course of the disease. The number of principal components (PCs) was determined using the Kaiser-Guttman criterion (eigenvalues >1). We complemented it with additional approaches, including visual inspection of the scree plot and the cumulative explained variance criterion. To facilitate interpretation, we use an orthogonal rotation (varimax). The loadings of each variable were determined algebraically, with each factor estimated as a linear combination of observed variables to achieve the best fit. Importantly, all included variables had communalities >0.9, except for PHR (0.326) and average CRP (0.654), which led to their removal. Finally, the PCA selected comprised 8 original inflammation-related variables obtained at the cut-off (i.e. PLR, NLR, MLR, SII, PIV, serum CRP at cut-off, CRP/albumin, and CRP/prealbumin), which were represented in 2 principal components (PCs) accounting for 95.3% of the variance: principal component 1 (PC-1) loaded the 6 variables based on the hematologic indices and ratios (i.e. NLR, PLR, MLR, SII, and PIV), whereas PC-2 loaded variables derived from cross-sectional serum CRP (CRP, CRP/albumin, and CRP/prealbumin).

**Figure 1:**
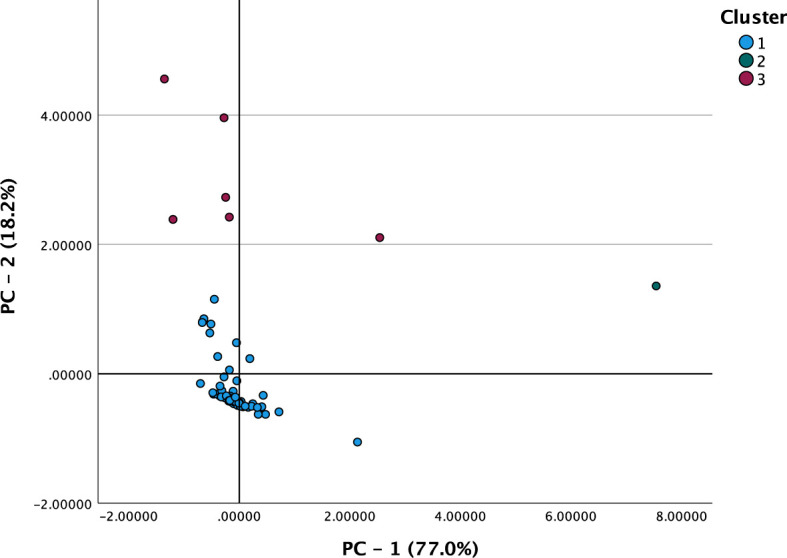
Component plot in rotated space with the original variables and the principal components.

The two PCs were used for k-means clustering, resulting in three groups (**Figure 1**): cluster 1 comprised 72 patients with a low-moderate inflammatory profile in both PCs; cluster 2 comprised a single patient with an extreme inflammatory profile; and cluster 3 comprised 6 patients with a moderate inflammatory profile in PC-1 and a high profile in PC-2. Multiple random centroids were run with a maximum iteration of 10. The algorithm reached convergence in 3 iterations. The differences between the clusters were validated using ANOVA, which yielded very high F values ​​for both PCs (p < 0.001). Because cluster 2 consists of an unrepresentative case, it was decided to regroup it into the closest cluster. After this modification, we re-evaluated the association of the new clusters with key clinical outcomes. The results remained consistent, with the Mann-Whitney test showing p=0.501 for PC-1 and p<0.001 for PC-2, indicating that the biological interpretation of the findings was not compromised by the adjustment.

Finally, binary logistic regression models were used to identify possible independent factors associated with the dependent variables, as follows: persistent inflammatory phenotype, inflammatory cluster derived from the k-means test, ILD, and respiratory damage associated with SSc. The statistical analyses were performed using IBM SPSS Statistics for Mac OS, Version 28 (IBM Corp., Armonk, NY, USA), licensed to staff of the University of Malaga.

## 3 Results

### 3.1 General characteristics of patients with SSc

The study sample comprised 83 patients with SSc. **Table 1** shows the sociodemographic, clinical, and therapy-related characteristics. Mean age was 58.6 years (range 20 – 87), and most patients were women (96.4%) and of European origin (86.7%). The socioeconomic level was low (51.8%). With respect to smoking, 62.7% had never smoked, 37.3% had smoked but given up, and only 7.2% continued to smoke. The age-adjusted CCI showed that comorbid disease burden was greater in patients with SSc as a whole (median, 3), although their cardiovascular risk was low according to SCORE (median [IQR], 1.0 [2.0]). The most frequent comorbidity was dyslipidemia (28.9%), followed by arterial hypertension (25.3%) and hypothyroidism (15.7%).

**Table 1:** General characteristics of the 83 patients with SSc.

Variables	N = 83
Socio-demographics	
Age, mean (SD), yrs	58.6 (10.7)
Women, n (%)	80 (96.4)
Ethnicity, White, n (%)	72 (86.7)
Education level, n (%)	
Primary or no education	47 (56.6)
Secondary	25 (30.1)
University	11 (13.3)
Economic income, €/month	
No income	19 (22.9)
<1,500	48 (57.8)
≥1,500	16 (19.3)
Smoking status, n (%)	
Never smoker	52 (62.7)
Ever smoker	31 (37.3)
Current smoking	6 (7.2)
Past smoking	25 (30.1)
Comorbidity	
Age-CCI, median (IQR)	3.0 (2.0)
SCORE, median (IQR)	1.0 (2.0)
Clinical characteristics	
SSc duration, median (IQR), yrs	9.0 (12.0)
SSc duration ≤5 yrs	29 (34.9)
Disease Classification, n (%)	
lSSc	1 (1.2)
lcSSc	67 (80.7)
dcSSc	15 (18.1)
Skin thickening of the fingers of both hands extending proximal to MCP joints, n (%)	15 (20.0)
Puffy fingers, n (%)	33 (39.8)
Sclerodactyly, n (%)	65 (78.3)
Digital tip ulcers, n (%)	20 (24.1)
Fingertip pitting scars n (%)	7 (8.4)
Telangiectasia, n (%)	71 (85.5)
Abnormal nail capillaries, n (%)	70 (84.3)
Raynaud’s phenomenon, n (%)	83 (100)
Arthritis, n (%)	15 (18.1)
Tendon friction rubs, n (%)	4 (4.8)
Calcinosis, n (%)	25 (30.1)
Gastrointestinal manifestations	74 (89.2)
Microstomy	44 (53.0)
Xerostomia	22 (26.5)
Gastroesophageal reflux	50 (60.2)
Primary biliary cholangitis	4 (4.8)
Dysphagia	42 (50.6)
Esophageal dysmotility	8 (9.6)
Abdominal distension/swelling	42 (50.6)
ILD, n (%)	28 (33.7)
HRCT NSIP pattern, n (%)	21 (25.3)
HRCT fibrotic NSIP pattern, n (%)	5 (6.0)
HRCT UIP pattern, n (%)	1 (1.2)
Pulmonary Function Testing at protocol	
SatO2, media (SD), %	97 (1.8)
FVC, media (SD), %	82.1 (20.6)
FEV1, media (SD), %	85.9 (20.1)
FEV1/FVC, media (SD), %	106.6 (15.1)
DLCO, media (SD), %	66.6 (21.2)
KCO, media (SD), %	80.1 (18.8)
FVC/DLCO, media (SD)	1.3 (0.4)
PASP (echocardiography), media (SD), mmHg	33.3 (9.5)
PAH, n (%)	7 (8.4)
Pericarditis, n (%)	1 (1.2)
Autoantibody profile, n (%)	
Antinuclear antibodies	80 (96.4)
Anti-centromere proteins	47 (56.6)
Anti-Scl70+	18 (21.7)
Anti-RNA polymerase III	2 (2.4)
Anti-PM/Scl	4 (4.8)
Anti-Ku	2 (2.4)
Anti-Ro/SS-A	10 (12.0)
Anti-U1-RNP	4 (4.8)
Treatments	
Methotrexate, n (%)	39 (47)
Hydroxychloroquine, n (%)	15 (18.1)
Mycophenolate, n (%)	23 (27.7)
Cyclophosphamide, n (%)	4 (4.8)
Calcium Channel Blockers, n (%)	63 (75.9)
PDE5 inhibitors, n (%)	15 (18.1)
Endothelin receptor antagonists, n (%)	11 (13.3)
ACE inhibitors, n (%)	11 (13.3)
Antifibrotic, n (%)	3 (3.6)
Rituximab, n (%)	4 (4.8)
Tocilizumab, n (%)	2 (2.4)
Prednisone, n (%)	41 (49.4)

Abbreviations: SCTC DI: Scleroderma Clinical Trials Consortium Damage Index; IQR: interquartile range; BMI: Body Mass Index; Age-CCI: age-adjusted Charlson Comorbidity Index; SCORE, European risk assessment score. EQ-5D: EuroQol Group-5D Questionnaire; VAS EQ-5D: Visual Analogue Scale EuroQol Group-5D Questionnaire; lSSc: Limited Systemic Sclerosis; lcSSc: Limited Cutaneous Systemic Sclerosis; dcSSc: Diffuse Cutaneous Systemic Sclerosis; MCP: Metacarpophalangeal; HRCT: High-Resolution Computed Tomography; ID: Interstitial Lung Disease; NSIP: Nonspecific interstitial pneumonia; UIP: Usual interstitial pneumonia; PASP: pulmonary arterial systolic pressure. PAH: pulmonary arterial hypertension; mRSS: Modified Rodnan Skin Score;CRP: C-reactive protein; PDE5I: phosphodiesterase 5 inhibitor.

The median disease duration from onset of the first non-Raynaud symptoms was 9 years; one-third of the sample had a disease duration of less than 5 years. As for skin involvement spread, the main subtype was lcSSc (80.7%), and the median MRSS was 8. Regarding SSc-related skin lesions, Raynaud phenomenon was universally present, followed in frequency by cutaneous sclerosis (98.8%) and telangiectasia (85.5%). Other than the skin, the most frequently affected organ system was the digestive tract (89.2%), followed by the respiratory system. SSc-ILD affected 1 in 3 patients, with the predominant radiological pattern being NSIP. Average PFT values remained above 80%, except for DLCO, which was the most affected. All patients with SSc but 3 had positive ANA values, with significant titers. The predominant specific antibodies were anticentromere antibodies, followed by anti-Scl70 and other, less common specific types.

Methotrexate was the most frequently prescribed immunosuppressant from disease onset (47%). Patients who did not respond to or experienced adverse effects with methotrexate were treated with alternative immunosuppressants, most commonly mycophenolate mofetil (27.7%), followed by cyclophosphamide or biologic agents. Specifically, four patients received rituximab, and one patient received tocilizumab. In total, twenty-four patients (28.9%) required an immunosuppressant other than methotrexate, which included mycophenolate mofetil, cyclophosphamide, and biologic agents. Forty-one patients (49.4%) were taking or had taken low-dose glucocorticoids (prednisone equivalents ≤10 mg/d). The most frequently prescribed vasodilators were calcium antagonists (75.9%), followed by sildenafil (18.1%). Eleven patients (13.3%) took endothelin receptor antagonists and 3 (3.6%) took antifibrotics.

**Table 2** shows all the parameters related to SSc-induced damage according to the SCTC-DI values, blood counts, and variables associated with serum CRP. Notably, CRP levels were elevated (≥5 mg/L) in 30.1% of patients.

**Table 2:** Variables related to inflammation and damage in SSc patients.

Variables	N = 83
MRSS, median (IQR)	8.0 (10.0)
SCTC DI, median (IQR)	3.0 (6.0)
Musculoskeletal and skin, n (%)	32 (38.6)
Vascular, n (%)	16 (19.3)
Gastrointestinal, n (%)	36 (43.4)
Respiratory, n (%)	25 (30.1)
Cardiovascular, n (%)	10 (12.0)
Renal, n (%)	2 (2.4)
Cell counts and ratios	
Hemoglobin, median (IQR), g/dL	12.8 (1.3)
Leucocytes, median (IQR), 109/mL	6.8 (3.2)
Neutrophils, median (IQR), 10^9^/mL	4.2 (2.6)
Lymphocytes, median (IQR), 10^9^/mL	1.6 (0.8)
Monocytes, median (IQR), 10^9^/mL	0.5 (0.3)
Platelets, media (SD), 10^9^/mL	253.0 (86.0)
Neutrophils to Lymphocytes ratio, median (IQR)	2.4 (1.6)
Monocytes to Lymphocytes ratio, median (IQR)	0.3 (0.2)
Platelets to Lymphocytes radio, median (IQR)	147.5 (73.0)
Platelets to Hemoglobin ratio, media (SD)	194.2 (81.4)
Systemic inflammation immune index, median (IQR)	560.3 (461.4)
Pan-immune inflammation values, median (IQR)	320.1 (366.0)
Serum CRP at protocol, median (IQR), g/L	4.0 (1.0)
Serum CRP ≥5mg/L at protocol, n (%)	25 (30.1)
CRP/albumin ratio, median (IQR)	1.0 (0.4)
CRP/prealbumin ratio, median (IQR)	0.2 (0.1)
Serum CRP average during follow-up, median (IQR), g/L	4.3 (4.2)
Serum CRP averaged ≥5mg/L, n (%)	35 (42.2)
Number of visits with CRP >5mg/L, median (IQR)	1.0 (4.0)
Percentage of visits with CRP >5mg/L, median (IQR)	11.1 (40.0)
Duration of observation with PCR, median (IQR), years	3.7 (1.9)
Inflammatory phenotypes during follow-up	
Non-inflammatory, n (%)	50 (60.2)
Intermediate, n (%)	25 (30.1)
sPersistent, n (%)	8 (9.6)

Abbreviations: MRSS: modified Rodnan skin score; SCTC DI: Scleroderma Clinical Trials Consortium Damage Index; IQR: interquartile range; CRP: C reactive protein.

### 3.2 Association between inflammation and characteristics of patients with SSc

**Figure 2:**
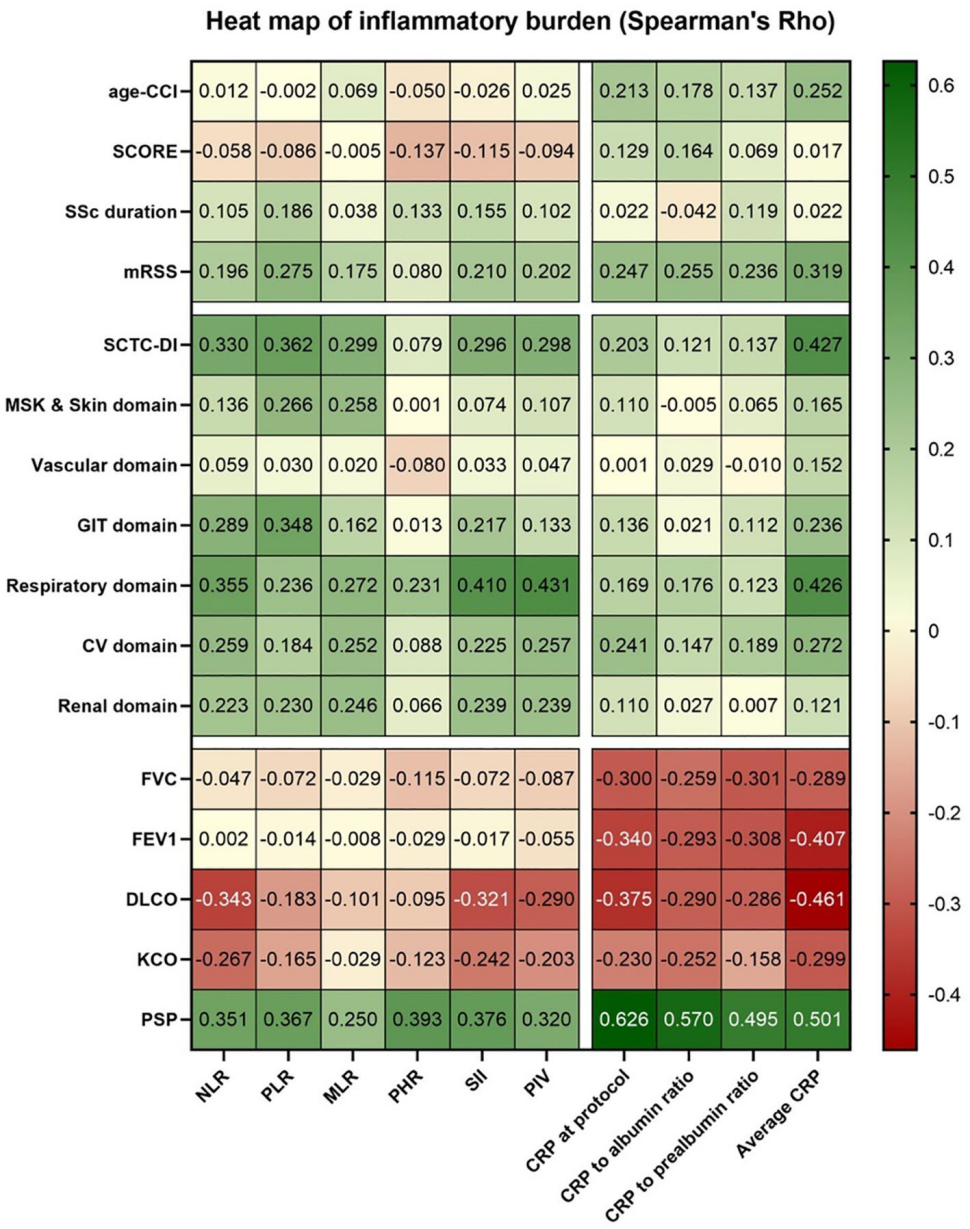
Heat map showing correlations for inflammatory burden in 83 patients with SSc.

**Figure 2** and **Supplementary Tables 1** and **2** present the correlations between inflammatory markers and respiratory damage. Hemoglobin values were not significantly correlated with blood counts or serum CRP, except for PHR (rho –0.340; p=0.002). The strongest correlations between serum CRP and blood counts were observed for average CRP and PIV (rho 0.454; p<0.001), neutrophils (rho=0.435; p<0.001), and leukocytes (rho 0.432; p<0.001).

**Supplementary Table 2** shows that age-adjusted CCI correlated weakly with average CRP (rho 0.252; p=0.022). Significant associations were found between inflammatory markers and disease burden, with moderate correlations between average CRP and MRSS (rho 0.319; p=0.003), as well as SCTC-DI and several inflammatory indices (NLR, PLR, MLR, SII, PIV, CRP at the cut-off, and average CRP). Among damage domains, the strongest correlations were observed in the respiratory domain. FVC showed moderate negative correlations with CRP at the cut-off and average CRP.

**Supplementary Table 3** summarizes the bivariate analyses. Statistically significant differences were found for smoking, with higher PIV values in smokers (p=0.033), and for ethnicity, with non-Caucasian patients showing higher CRP levels. Anti-Scl70 positivity was associated with increased inflammatory markers, while anticentromere positivity was linked to lower PHR, SII, and PIV values. ILD and respiratory damage were primarily associated with blood count-based parameters.

Patients receiving glucocorticoids and immunosuppressants, particularly biologics or non-methotrexate drugs, had significantly higher inflammatory marker levels.

### 3.3 Characteristics of patients with SSc by degree and type of inflammation

#### 3.3.1 Stratification by inflammatory phenotypes based on average CRP during follow-up

During the course of the disease, we collected serum CRP values from 83 patients with SSc at 822 consecutive visits before the cut-off, that is, a total follow-up of 318.9 patient-years and a median (IQR) of 9.6 (3) visits (range, 4 – 19) per patient. At the cut-off, the median CRP was 4 mg/L (range, 4 – 39), with 42.2% of patients showing elevated levels at previous visits. According to these average CRP values during follow-up and the cut-off values applied by EUSTAR, most patients had a noninflammatory pattern (60.2%), followed by an intermediate pattern (30.1%), and a persistent pattern (9.6%).

**Table 3** shows the patients’ characteristics stratified by the 3 inflammatory phenotypes defined by EUSTAR during the course of the disease. According to these phenotypes, patients with SSc did not differ significantly in age, disease duration, sex, socioeconomic level, smoking, alcohol consumption, age-adjusted CCI, risk according to SCORE, extension and severity of the skin condition (MRSS), or time under observation during follow-up of the CRP values. While not a statistically significant difference (p=0.065), the percentage of Caucasian patients decreased as the inflammatory phenotype progressed.

**Table 3:** Características de los pacientes estratificados por fenotipos inflamatorios.

	Non-inflammatory phenotype (N=50)	Intermediate inflammatory phenotype (N=25)	Persistent inflammatory phenotype (N=8)	*p*-value	Post-hoc
Age, media (SD), yrs	57.8 (8.8)	59.6 (14.9)	60.4 (7.0)	0.621	
Sex, Women, n (%)	48 (96)	25 (100)	7 (87.5)	0.250	
Ethnicity, Caucasian, n (%)	46 (92.0)	21 (84.0)	5 (62.5)	0.065	
Ever smoked, n (%)	16 (32.0)	12 (48.0)	3 (37.5)	0.402	
Alcohol, n (%)	2 (4)	2 (8)	1 (12.5)	0.569	
Low socioeconomic level	26 (52.0)	12 (48.0)	5 (62.5)	0.774	
Age-CCI, median (IQR)	2.0 (1.0)	3.0 (3.0)	2.5 (2.0)	0.184	
SCORE, median (IQR)	1.0 (2.0)	1.0 (2.0)	2.0 (2.0)	0.562	
Duration of SSc, median (IQR)	10.0 (11.5)	9.0 (10)	6.0 (17.5)	0.877	
Disease duration ≤5 years, n (%)	16 (32)	9 (36)	4 (50)	0.606	
Duration of CRP observation, median (IQR), yrs	3.7 (2.0)	3.7 (1.9)	2.9 (1.8)	0.430	
Disease Classification					
lcSSc, n (%)	43 (86.0)	15 (62.5)	7 (87.5)	0.055	
dcSSc, n (%)	5 (10.2)	8 (32)	1 (12.5)	0.058	
MRSS	6.0 (8.0)	8.0 (16.0)	12.0 (11.0)	0.093	
Anemia, n (%)	7 (14)	6 (24)	3 (37.5)	0.228	
Puffy fingers, n (%)	17 (34)	12 (48)	4 (50)	0.417	
Sclerodactyly, n (%)	36 (72)	23 (92)	6 (75)	0.137	
Digital tip ulcers, n (%)	11 (22)	7 (28)	2 (25)	0.847	
Abnormal nail capillaries, n (%)					
Dilated nail capillaries, n (%)	47 (100)	17 (85)	6 (100)	0.016	NIP>IIP*
Low capillary density, n (%)	18 (38.3)	13 (65)	3 (50)	0.132	
Arthralgia, n (%)	31 (62)	20 (80)	6 (75)	0.263	
Arthritis, n (%)	7 (14)	5 (20)	3 (37.5)	0.264	
Tendon friction rubs, n (%)	3 (6)	1 (4)	0 (0)	0.743	
Myalgia, n (%)	1 (2)	4 (16)	0 (0)	0.042	
Calcinosis, n (%)	11 (22)	13 (52)	1 (12.5)	0.015	IIP>PIP*
Persistent digestive symptoms, n (%)	45 (90)	21 (84)	8 (100)	0.428	
Renal crisis, n (%)	1 (2)	1 (4)	0 (0)	0.778	
Pericarditis, n (%)	0 (0)	1 (4)	0 (0)	0.309	
Dyspnea, n (%)	13 (26)	9 (36)	7 (87.5)	0.003	PIP>NIP*
SSc-ILD, n (%)	10 (23.3)	10 (43.5)	6 (75)	0.011	PIP>NIP*
NSIP, n (%)	7 (16.3)	10 (43.5)	4 (50)	0.023	PIP>NIP*
Fibrotic NSIP, n (%)	2 (4.7)	0 (0)	3 (37.5)	<0.001	PIP>IIP>NIP*
UIP, n (%)	1 (2.3)	0 (0)	0 (0)	0.694	
HAP, n (%)	4 (8.2)	2 (8)	1 (12.5)	0.914	
PSP, media (SD), mmHg	29.1 (6.9)	35.4 (7.0)	47.7 (8.6)	<0.001	PIP>NIP** / PIP>IIP*
Respiratory Function Tests					
FVC, media (SD), %	85.4 (20.7)	83.3 (13.62)	57.7 (19.7)	0.001	NIP>PIP** / IIP>PIP*
FEV1, media (SD), %	90.8 (18.8)	85.5 (14.8)	61.5 (22.7)	<0.001	NIP>PIP** / IIP>PIP*
FEV1/FVC, media (SD), %	108.1 (17.2)	103.1 (10.4)	107.5 (13.5)	0.437	
DLCO, media (SD), %	69.6 (20.3)	65.8 (21.1)	44.9 (12.4)	0.015	NIP>PIP* / IIP>PIP
KCO, media (SD), %	82.0 (17.5)	80.2 (19.4)	62.4 (14.8)	0.032	NIP>PIP* / IIP>PIP
FVC/DLCO, media (SD)	1.3 (0.4)	1.3 (0.4)	1.4 (0.3)	0.666	
SCTC DI, median (IQR)	2 (4.25)	4 (4.5)	6 (10.5)	0.022	PIP>NIP*
Weighted MSK & skin domain, median (IQR)	0.0 (3.0)	0 (2.5)	2.5 (3.0)	0.296	
Weighted Vascular domain, median (IQR)	0.0 (0.0)	0.0 (1.0)	0.0 (0.0)	0.726	
Weighted Gastrointestinal domain, median (IQR)	0.0 (1.0)	0.0 (1.0)	1.0 (1.7)	0.124	
Weighted Respiratory domain, median (IQR)	0.0 (0.0)	0.0 (4.0)	2.0 (5.7)	0.046	
Weighted CV domain, median (IQR)	0.0 (0.0)	0 .0 (0.0)	1.0 (2.0)	0.002	PIP>IIP** / PIP>NIP*
Weighted Renal domain, median (IQR)	0.0 (0.0)	0.0 (0.0)	0.0 (0)	0.780	
SCTC >= 13, n (%)	1 (2.0)	1 (4.0)	2 (25.0)	0.018	PIP>NIP
ANA, n (%)					
Anti-centromere proteins, n (%)	30 (60)	14 (56)	3 (37.5)	0.490	
Anti-Scl70+, n (%)	8 (16)	6 (24)	4 (50)	0.090	
Anti-PM/Scl, n (%)	4 (8.9)	0 (0)	0 (0)	0.234	
Anti-Ku, n (%)	1 (2.2)	1 (4.2)	0 (0)	0.803	
Anti-U1-RNP, n (%)	1 (2.2)	2 (8)	1 (14.3)	0.304	
Treatment					
Immunosuppressors, n (%)	33 (66)	16 (64)	8 (100)	0.131	
Non-methotrexate IS, n (%)	12 (24)	6 (24)	6 (75)	0.010	PIP>IIP>NIP*
Methotrexate, n (%)	24 (48)	11 (44)	4 (50)	0.933	
Hydroxychloroquine, n (%)	12 (24)	3 (12)	0 (0)	0.167	
Mycophenolate mofetil, n (%)	11 (22)	6 (24)	6 (75)	0.007	PIP>IIP>NIP*
Cyclophosphamide, n (%)	1 (2)	1 (4)	2 (25)	0.018	PIP>NIP*
Antifibrotic, n (%)	1 (16.7)	0 (0)	2 (100)	0.049	
Rituximab, n (%)	2 (4.1)	1 (4)	1 (12.5)	0.574	
Tocilizumab, n (%)	0 (0)	2 (8)	0 (0)	0.093	
Prednisone, n (%)	19 (38)	14 (56)	8 (100)	0.004	PIP>NIP*
Calcium Channel Blockers, n (%)	37 (74)	20 (80)	6 (75)	0.847	
PDE5 inhibitors, n (%)	7 (14.0)	6 (24.0)	2 (25.0)	0.493	
Endothelin receptor antagonists, n (%)	5 (10)	5 (20)	1 (12.5)	0.483	
ACE inhibitors, n (%)	6 (12)	4 (16.7)	1 (12.5)	0.856	
Neutrophils, median (IQR), 10^9^/mL	3.9 (2.1)	5.3 (2.3)	6.2 (10.4)	0.010	NIP>IIP*
Lymphocytes, median (IQR), 10^9^/mL	1.5 (0.7)	1.8 (2.1)	1.9 (1.3)	0.457	
Monocytes, median (IQR), 10^9^/mL	0.5 (0.2)	0.7 (0.4)	0.7 (0.4)	0.033	NIP>PIP*
Platelets, media (SD), 10^9^/mL	228 (64.56)	270.36 (67.66)	328 (107.53)	<0.001	PIP>NIP*
Neutrophils to Lymphocytes ratio, median (IQR)	2.2 (1.0)	2.8 (2.1)	3.7 (6.8)	0.033	
Monocytes to Lymphocytes ratio, median (IQR)	0.3 (0.1)	0.4 (0.2)	0.4 (0.2)	0.199	
Platelets to Lymphocytes radio, median (IQR)	137.2 (49.2)	156.7 (79.7)	226.2 (169.5)	0.251	
Platelets to Hemoglobin ratio, media (SD)	176.7 (52.5)	212.3 (57.6)	258.3 (98.5)	0.017	IIP>NIP*
Systemic inflammation immune index, median (IQR)	516.0 (357.3)	775.5 (702.9)	1192.4 (3463.7)	0.010	NIP>IIP*
Pan-immune inflammation values, median (IQR)	235.6 (274.9)	533.6 (455.2)	832.4 (1905.7)	0.003	NIP>IIP*

NIP: non-inflammatory phenotype; IIP: Intermediate inflammatory phenotype; PIP: Persistent inflammatory phenotype; MSK: musculoskeletal; IS: immunosuppressants. PASP, pulmonary arterial systolic pressure; PAH, pulmonary arterial hypertension; mRSS, modified Rodnan Skin Score; CRP, C-reactive protein; PDE5I, hosphodiesterase 5 inhibitor.

However, capillaroscopy did reveal significant differences in the capillary dilatation pattern (p=0.016), lung involvement, treatment administered, and cumulative damage (SCTC-DI) (p<0.011). More specifically, SSc-ILD was more frequent in patients with the persistent inflammatory phenotype (p=0.011), particularly NSIP (p=0.023) and fibrotic NSIP (p=0.001). Dyspnea was also more frequent in this group (p<0.001). In addition, the results of the PFT performed at the cut-off and PASP measured using transthoracic echocardiography were poorer (**Table 3**). Similarly, these patients were characterized by greater global damage (SCTC-DI) (p=0.011) and in the weighted domains of cardiovascular injury (p=0.001) and respiratory damage (p=0.022). As for the treatments administered, differences were observed for glucocorticoids (p=0.004), mycophenolate mofetil (p=0.007), intravenous cyclophosphamide (p=0.018), and antifibrotic agents (p=0.049).

When only patients with SSc-ILD were considered (N=26), the results of the PFT and PASP were poorer in those with the persistent inflammatory phenotype than in the others. Despite numerical differences in the treatments received by this group according to their inflammatory phenotype, these did not reach statistical significance because of the low number of patients.

### 3.4 Principal component analysis and clustering of the cross-sectional inflammatory variables

Two PCs with an eigenvalue >1 were extracted (**Supplementary Table 4**). The PCA selected two PCs with eigenvalues >1. These PCs comprised 8 original inflammation-related variables obtained at the cut-off (i.e. PLR, NLR, MLR, SII, PIV, serum CRP at cut-off, CRP/albumin, and CRP/prealbumin), which 95.3% of the cumulative variance: principal component 1 (PC-1) loaded the 6 variables based on the hematologic indices and ratios (i.e. NLR, PLR, MLR, SII, and PIV), whereas PC-2 loaded variables derived from cross-sectional serum CRP (CRP, CRP/albumin, and CRP/prealbumin). The 2 PCs described above were subsequently used to perform k-means clustering, which yielded 3 clusters (**Figure 1**): cluster 1 comprised 72 patients with a low-moderate inflammatory profile in both PCs; cluster 2 comprised a single patient with an extreme inflammatory profile; and cluster 3 comprised 6 patients with a moderate inflammatory profile in PC-1 and a high profile in PC-2. The similarities between the means of the 2 PCs within each of the clusters supports the notion that both PC-1 and PC-2 are capturing similar phenomena, albeit it reflects distinct biological processes (i.e., cellular vs. humoral inflammation) and with different degrees of sensitivity.

Comparison of the 3 cross-sectional groups using k-means clustering and the 3 longitudinal inflammatory phenotypes based on the average CRP values during follow-up revealed a significant association between both classifications, albeit with low agreement (kappa, 0.243; p<0.001).

Clusters 2 and 3 were collapsed into a single inflammation category for the clinical association analysis. Consequently, we obtained an additional variable with 2 categories: the noninflammatory group (n=72) and the inflammatory group (n=7). **Supplementary Table 5** shows the main patient characteristics stratified according to these 2 new groups. We can see that the association profiles for the cross-sectional groups were consistent with the 3 longitudinal inflammation phenotypes based on the average CRP values shown in **Table 3**. The only noteworthy difference was that the cluster-based inflammatory group was also associated with damage in the gastrointestinal domain (p=0.033), cardiovascular domain (p=0.007), and renal domain (p=0.039).

### 3.5 Factors associated with cross-sectional and longitudinal inflammatory phenotypes, SSc-ILD, and respiratory damage

**Table 4** shows the results of the multivariate binary logistic regression analysis with the persistent inflammatory phenotype as the dependent variable after adjusting for age, respiratory damage, and use of immunosuppressants other than methotrexate. Consistent with this analysis, and compared with the noninflammatory phenotype, the persistent inflammatory phenotype was associated with non-Caucasian ethnicity (OR 14.0) and with SSc-ILD (OR 17.9) (**Table 4**). Given that almost all the patients were women, the model was not adjusted for sex.

**Table 4:** Factors associated with persistent inflammatory phenotype (binary logistic regression analysis).

Predictor	Univariate			Multivariate		
	OR	95% CI	P-value	OR	95% CI	P-value
Age, years	1.0	0.9–1.1	0.415			
Non-Caucasian Ethnicity	6.9	1.2–40.0	0.031	14.0	1.2 – 166.1	0.037
SSc-ILD	9.9	1.7–56.9	0.010	17.9	1.9 – 169.3	0.012
Respiratory domain damage (weighted)	3.5	0.8–16.5	0.107			
Non-MTX immunosuppressant	9.2	1.6–52.0	0.012			

Variables specified in step 1: Age, Non-Caucasian Ethnicity, SSc-ILD, Weighted Respiratory domain damage, and non-MTX Immunosuppressants. Hosmer-Lemeshow test: 0.660; The model classifies 88.2% of the observed values; R^2^ Nagelkerke: 0.387.

95% CI, 95% confidence interval; MTX, Methotrexate.

**Table 5** shows the results of the multivariate binary logistic regression analysis with inflammatory clusters as the dependent variable. We can see that the cross-sectional inflammatory group was associated with SSc-ILD (OR 12.8) and organ damage overall (SCTC-DI) (OR 1.2) after adjusting for age, ethnicity, cumulative disease-induced damage in the respiratory and digestive systems, and immunosuppressants other than methotrexate.

**Table 5:** Factors associated with the cross-sectional inflammatory group (binary logistic regression analysis).

Predictor	Univariate			Multivariate		
	OR	95% CI	P-value	OR	95% CI	P-value
Age, years	1.0	0.9 – 1.1	0.541			0.759
Non-Caucasian Ethnicity	6.0	1.1 – 31.8	0.035			0.156
SSc-ILD	16.2	1.8 – 144.9	0.013	12.8	1.3 – 126.0	0.028
SCTC-DI	1.2	1.1 – 1.5	0.003	1.2	1.0 – 1.4	0.029
Respiratory domain damage (weighted)	1.5	1.1 – 1.9	0.009			0.740
Gastrointestinal domain damage (weighted)	1.7	0.8 – 3.6	0.137			0.776
Non-MTX immunosuppressant	9.2	1.6–52.0	0.012			0.945

Variables specified in step 1: Age, Non-Caucasian Ethnicity, SSc-ILD, Weighted Respiratory domain damage, Weighted Gastrointestinal domain damage, SCTC-DI, and non-MTX Immunosuppressants. Hosmer-Lemeshow test: 0.414; The model classifies 90.0% of the observed values; R^2^ Nagelkerke: 0.400.

95% CI, 95% confidence interval; SCTC-DI, Scleroderma Clinical Trials Consortium Damage Index; MTX, Methotrexate.

**Tables 6** and **7** show the association between SSc-ILD and SSc-related cumulative damage in the respiratory domain, with the inflammation-related variables using the components obtained in the PCA and average CRP observed during follow-up and adjusted for other factors and confounders such as age, ethnicity, anti-Scl70+, and treatment with immunosuppressants or corticosteroids.

**Table 6:** Factors associated with SSc-ILD (binary logistic regression analysis).

Predictor	Univariate			Multivariate		
	OR	95% CI	P-value	OR	95% CI	P-value
Age, years	1.0	0.9 – 1.1	0.492			0.184
Non-Caucasian Ethnicity	1.1	0.3 – 4.0	0.926			0.193
Anti-Scl70+	2.4	5.0 – 83.2	<0.001	19.1	2.2 – 162.4	0.007
PC-1	0.9	0.5 – 1.5	0.694	0.5	0.2 – 1.2	0.116
PC-2	2.2	1.2 – 4.2	0.011	3.0	1.1 – 8.0	0.026
Average CRP, mg/L	1.2	1.0 – 1.3	0.016			0.848
Non-MTX immunosuppressant	33.7	8.0 – 141.7	<0.001	42.2	5.7 – 311.9	<0.001

Variables specified in step 1: Age, non-Caucasian Race, anti-Scl70+, PC-1, PC-2, average CRP, and non-MTX immunosuppressants. Hosmer-Lemeshow test: 0.170; The model classifies 88.6% of the observed values; R^2^ Nagelkerke: 0.689.

95% CI, 95% confidence interval; PC-1, Principal Component 1 (related to the 6 hematological variables); PC-2, Principal Component 2 (related to the 3 variables related to serum PCR at protocol); MTX, Methotrexate.

**Table 7:** Factors associated with SCTC-DI respiratory domain damage (binary logistic regression analysis).

Predictor	Univariate			Multivariate		
	OR	95% CI	P-value	OR	95% CI	P-value
Age, years	1.0	0.9 – 1.1	0.363			0.257
Non-Caucasian Ethnicity	3.3	0.9 – 12.2	0.068			0.530
Anti-Scl70+	3.1	1.0 – 9.0	0.043	7.7	1.5 – 38.6	0.013
PC-1	1.9	0.8 – 4.4	0.151			0.089
PC-2	1.5	0.9 – 2.4	0.090			0.607
Average CRP, mg/L	1.1	1.0 – 1.3	0.013	1.2	1.0 – 1.4	0.027
Glucocorticoids	0.9	0.4 – 2.4	0.867	0.2	0.1 – 0.9	0.046

Variables specified in step 1: Age, non-Caucasian Race, anti-Scl70+, PC-1, PC-2, average CRP, and Glucocorticoids. Hosmer-Lemeshow test: 0.463; The model classifies 74.7% of the observed values; R^2^ Nagelkerke: 0.279.

SCTC-DI, Scleroderma Clinical Trials Consortium Damage Index; 95% CI, 95% confidence interval; PC-1, Principal Component 1 (related to the 6 hematological variables); PC-2, Principal Component 2 (related to the 3 variables related to serum PCR at protocol).

In line with the results presented in **Table 6**, PC-2 (associated mainly with CRP at the cut-off and its variants) (OR 3.0), but not PC-1 (associated mainly with blood counts), was associated with SSc-ILD in the presence of anti-Scl70+ (OR 19,1) and immunosuppressive treatment (OR 42.2).

In contrast (see **Table 7**), none of the PCA components were selected as factors associated with respiratory damage factors when the model was adjusted for average CRP during follow-up (OR 1.2), anti-Scl70+ (OR 7.7), and treatment with glucocorticoids, which showed a protective effect (OR 0.2).

## 4 Discussion

In our study, high CRP levels were recorded in 30.1% of patients at baseline and in 42.2% during follow-up, with around 10% exhibiting a persistently inflammatory phenotype. These findings suggest an association between chronic inflammation and SSc-ILD, reinforcing the hypothesis that inflammation plays a key role in respiratory damage. Additionally, our results support CRP as a more relevant marker than hematologic indices for predicting ILD and lung involvement in SSc.

The pathogenesis of SSc is based on three pillars: microvascular damage, immune dysregulation, and multiorgan fibrosis. Immune dysregulation involves cells, antibodies, and inflammatory signals in tissues such as the skin and lungs. Different sources of evidence highlight the importance of IL-6 in SSc, leading this protein to be investigated as a therapeutic target in patients with SSc-ILD (25). IL-6 induces CRP production in hepatocytes and activates immune and endothelial cells. In patients with SSc, IL-6 levels correlate strongly (rho 0.687) with serum CRP (26). However, persistently elevated CRP levels are uncommon in these patients. The prevalence of high CRP values in SSc ranges from 22.4% to 53.8% depending on the sample characteristics and methods used for measurement (26–29), with the highest values typically found in studies using hs-CRP or including patients with early disease (28, 30). In our study, no correlation was found between CRP and disease duration, consistent with the findings of Mitev et al. (31), potentially due to the impact of ongoing treatments (31–33).

Serum CRP has been proposed as a prognostic biomarker to guide decisions on therapy in daily clinical practice. Several studies, including a large-scale Canadian study and a study from EUSTAR, have reported an association between high serum CRP levels and involvement of the skin, joints, lungs, and kidneys (5, 29, 30, 32, 34, 35). The EUSTAR studies support the notion that a persistent inflammatory phenotype plays a role in the prognosis of patients with SSc, irrespective of demographics, disease duration, cutaneous subtype, and severity of SSc-ILD (5, 33). Consistent with the results of the Canadian and EUSTAR studies, we found that the persistent inflammatory phenotype was uncommon among our cohort and that it was associated mainly with SSc-ILD, poorer PFT results, greater PASP values in the echocardiogram, more pronounced cumulative damage according to the SCTC-DI, and a greater need for treatment, including corticosteroids, immunosuppressants, and antifibrotic agents. While other studies did not specifically analyze this phenotype as a dependent variable, our findings are consistent with those of most previous studies: patients with SSc-ILD and of non-Caucasian ethnicity were more likely to have the persistent inflammatory phenotype, irrespective of age, treatment received, and cumulative respiratory damage. This observation also reflects the impact of race and ethnicity as confounders, where biologic, socioeconomic, and cultural aspects combine to markedly affect inflammation and ILD (36, 37).

The connection between inflammation and ILD has been established in various IMIDs based on other markers of inflammation (38, 39). However, some parameters of nonspecific systemic inflammation, such as the indices and ratios based on peripheral blood counts have received less attention in SSc-ILD. Patients with SSc have been reported to present high levels of neutrophils, monocytes, and platelets, together with lower levels of lymphocytes and hemoglobin than healthy individuals. These indices and ratios are moderately correlated with serum CRP and could be associated with manifestations of SSc (e.g., vascular and cutaneous involvement), PFT findings, and greater mortality (12–14, 40). Nevertheless, findings for these markers remain limited, and no studies draw comparisons with CRP beyond simple correlations (10). Combined use of such markers could provide a more complete vision of inflammation in patients with SSc.

In this study, we compared both groups of inflammatory parameters (cell indices and CRP) to determine their relative weight in SSc-ILD. PCA was used to reduce colinearity and compact the variables into 2 dimensions that could capture most of the original variability so that they could then be used as factors in the multivariate models. The model showed that only CRP-derived variables (PC-2) were associated with SSc-ILD, whereas blood counts (PC-1) were excluded from the model. However, the magnitude of association for anti-Scl70+ and immunosuppressive treatment proved to be greater than for inflammation. Of note, immunosuppressive treatment could be reflecting a selection bias, since patients with more severe disease would be more likely to receive more potent immunosuppressants, thus potentially confounding interpretation of its impact in SSc-ILD.

A completely novel result of the present study has been the demonstration of an association between mean serum CRP and respiratory damage in patients with SSc. Furthermore, in the models evaluating respiratory damage, none of the principal components formed by cross-sectional inflammatory variables remained relevant, although average CRP regained relevance, thus highlighting its role as a marker of persistent inflammation in the long term, together with antibodies and glucocorticoids. This contrasts with markers of recent inflammation. Given this finding, the presence of elevated CRP together with SSc-ILD reflects current inflammatory activity, whereas irreversible damage requires persistent inflammation, as reflected by the average CRP value, which captures pathological processes in the long term. Moreover, glucocorticoids were used in <50% of patients, generally to treat inflammatory exacerbations in the early phases, which would a protective effect of glucocorticoids on progression of damage.

Our study is subject to a series of limitations. The main limitations are its observational design (noncontrolled cross-sectional), do not include healthy individuals, and adjusted sample size, which could hamper stratification of the subgroups most affected by inflammation and preclude us from establishing causal relationships. Nevertheless, our approach enabled us to cover the main objectives, namely, to perform an in-depth analysis of the sample and obtain results with high internal coherence. We also considered the average CRP levels collected throughout follow-up to better reflect chronic inflammation. One limitation of our clustering approach is that one of the initial clusters contained only a single patient, suggesting a potentially unstable solution. To address this, we merged clusters 2 and 3 into a single inflammatory group, ensuring a more robust classification. In addition, the sample comprised mainly women, thus precluding analysis by sex. However, this proportion reflects the distribution of patients with SSc in clinical practice. A greater number of men would have affected the external validity of the study. Similarly, recruiting all patients from a single center in Spain may limit the generalizability of our findings to other populations with different genetic and environmental backgrounds. However, the cohort reflects real-world characteristics of SSc in Southern Europe, providing valuable insights. The small sample size may also increase the risk of overfitting, particularly in the clustering analysis. Despite this, we identified significant differences in inflammatory profiles and their associations with key clinical outcomes, supporting the relevance of our findings. Moreover, while we detected signs of more marked chronic inflammation in non-Caucasian patients, this is not clearly reflected in the multivariate analyses owing to the low number of these patients. Furthermore, and of particular interest, is that a longitudinal analysis of CRP based on the average number of visits could mitigate the individual variability in inflammation over time, without capturing the real dynamics of inflammation. However, this was not our main objective. Finally, we recognize that both CRP and cell indices and ratios are nonspecific indicators of systemic inflammation. More in-depth assessment of chronic inflammation in the future would require longitudinal studies and other inflammatory biomarkers. Additionally, comparing CRP with established clinical models would be an improvement. However, since our objective was to evaluate the relationship of different inflammatory biomarkers with ILD, in future research we could explore more comprehensive prediction models. Furthermore, while we did not directly compare our models against established clinical prediction models, our multivariate analyzes included key clinical variables, and we presented only the most robust models in the results, while several alternative models were tested but not included.

Our study also has a series of strengths, such as the length of follow-up and the inclusion of multiple inflammatory parameters (both molecular and cellular). PCA helps to reduce complexity and enables more accurate assessment of inflammation.

In conclusion, our results show an association between inflammation and SSc-ILD and suggest that chronic inflammation could play a key role in the onset of respiratory damage. Our results also support the hypothesis that CRP is a more relevant marker than hematologic indices for predicting SSc-ILD and respiratory damage. Patients with anti-Scl70 positivity and inadequate response or intolerance to MTX may benefit from closer inflammatory monitoring, as they could require alternative therapeutic strategies. While hematologic indices could prove useful for detecting inflammation, they are less sensitive, especially in chronic disease. These results highlight the importance of continuous long-term monitoring of serum PCR, especially in patients with positive anti-Scl70 values who do not respond to or tolerate methotrexate. Similarly, they suggest that markers of inflammation could act as therapeutic targets in SSc-ILD. The development of composite biomarkers combining cellular and molecular approaches may improve our ability to predict disease progression and guide treatment.

## Data Availability

The original contributions presented in the study are included in the article/**Supplementary Material**. Further inquiries can be directed to the corresponding author.
