# Supplementary material for: Identifying inflammatory phenotypes associated with lung involvement in systemic sclerosis: k-means clustering approach
Source: Front Immunol. 2025 May 8;16:1568683. doi: 10.3389/fimmu.2025.1568683 (PMC12094983; doi:10.3389/fimmu.2025.1568683)
Supplement: Supplementary file 1 [file Table1.docx]

Supplementary Table 1. Correlations between inflammatory markers

|  | NLR | PLR | MLR | PHR | SII | PIV | CRP | CRP/albumin ratio | CRP/prealbumin ratio | Average CRP | Visits with CRP >5mg/L (%) |
| --- | --- | --- | --- | --- | --- | --- | --- | --- | --- | --- | --- |
| Hb | –0.200 | –0.170 | –0.026 | –0.340** | -0.106 | 0.012 | 0.007 | -0.170 | 0.080 | -0.132 | ­–0.112 |
| Neutrophils | 0.658** | 0.176 | 0.299* | 0.469** | 0.752** | 0.836** | 0.318* | 0.220* | 0.175 | 0.435** | 0.397** |
| Platelets | 0.216* | 0.436*** | 0.037 | 0.953** | 0.632** | 0.669** | 0.323* | 0.285* | 0.220* | 0.280* | 0.310* |
| Lymphocytes | –0.502** | –0.652** | –0.669** | 0.245* | 0.240* | –0.051 | 0.031 | 0.004 | 0.024 | 0.067 | 0.118 |
| Monocytes | 0.306** | 0.051 | 0.488** | 0.342* | 0.485** | 0.766** | 0.372** | 0.305* | 0.223* | 0.405** | 0.374** |
| NLR |  | 0.624** | 0.642** | 0.241* | 0.851** | 0.726** | 0.202 | 0.142 | 0.108 | 0.328* | 0.282* |
| PLR | 0.624** |  | 0.593** | 0.464** | 0.711** | 0.521** | 0.199 | 0.166 | 0.129 | 0.189 | 0.149 |
| MLR | 0.642** | 0.593** |  | 0.050 | 0.544** | 0.266* | 0.266* | 0.221* | 0.169 | 0.221* | 0.161 |
| PHR | 0.241* | 0.464** | 0.050 |  | 0.621** | 0.607** | 0.270* | 0.280* | 0.157 | 0.251* | 0.277* |
| SII | 0.851** | 0.711** | 0.544** | 0.621** |  | 0.922** | 0.316* | 0.224* | 0.190 | 0.381** | 0.360** |
| PIV | 0.726** | 0.521** | 0.576** | 0.607** | 0.922** |  | 0.410** | 0.305* | 0.264* | 0.454** | 0.426** |
| CRP, mg/L |  |  |  |  |  |  |  | 0.819** | 0.729** | 0.526** | 0.571** |
| CRP/albumin ratio |  |  |  |  |  |  | 0.819** |  | 0.606** | 0.533** | 0.551** |
| CRP/prealbumin ratio |  |  |  |  |  |  | 0.762** | 0.606** |  | 0.298** | 0.399** |
| Average CRP, mg/L |  |  |  |  |  |  | 0.26** | 0.533** | 0.298** |  | 0.882** |
| Percentage of visits with CRP >5mg/L |  |  |  |  |  |  | 0.571** | 0.551** | 0.393** | 0.882** |  |

Significance of Spearman’s Rho: *p<0.05; **p<0.001

Supplementary Table 2. Correlations between inflammatory markers and respiratory damage

|  | NLR | PLR | MLR | PHR | SII | PIV | CRP at protocol | CRP to albumin ratio | CRP to prealbumin ratio | Average CRP |
| --- | --- | --- | --- | --- | --- | --- | --- | --- | --- | --- |
| Age, yrs | –0.028 | –0.036 | 0.011 | –0.118 | –0.076 | –0.079 | 0.120 | 0.100 | 0.038 | 0.114 |
| SSc duration, yrs | 0.105 | 0.186 | 0.038 | 0.133 | 0.155 | 0.102 | 0.022 | -0.042 | 0.119 | 0.022 |
| Age-CCI | 0.012 | -0.002 | 0.069 | -0.050 | -0.26 | 0.025 | 0.213 | 0.178 | 0.137 | 0.252* |
| SCORE | -0.058 | -0.086 | -0.005 | -0.137 | -0.115 | -0.094 | 0.129 | 0.164 | 0.069 | 0.017 |
| MRSS | 0.196 | 0.275* | 0.175 | 0.080 | 0.210 | 0.202 | 0.247* | 0.255* | 0.236* | 0.319** |
| SCTC-DI total score | 0.286** | 0.309** | 0.288** | 0.090 | 0.286** | 0.320** | 0.246* | 0.150 | 0.158 | 0.365*** |
| MSK & Skin domain | 0.108 | 0.236* | 0.257* | 0.015 | 0.074 | 0.130 | 0.038 | 0.018 | 0.083 | 0.124 |
| Vascular domain | 0.055 | 0.022 | 0.013 | -0.087 | 0.027 | 0.042 | 0.005 | 0.026 | –0.013 | 0.152 |
| GIT domain | 0.285* | 0.313*** | 0.162 | 0.026 | 0.214* | 0.156 | 0.167 | 0.047 | 0.133 | 0.185* |
| Respiratory domain | 0.310*** | 0.193 | 0.266* | 0.242* | 0.407*** | 0.451*** | 0.205 | 0.204 | 0.146 | 0.364*** |
| CV domain | 0.209 | 0.137 | 0.244* | 0.102 | 0.217* | 0.280* | 0.274* | 0.177 | 0.210 | 0.204 |
| Renal domain | 0.223* | 0.230* | 0.246* | 0.066 | 0.239* | 0.239* | 0.110 | 0.027 | 0.007 | 0.121 |
| FCV% | -0.047 | -0.072 | -0.029 | -0.115 | -0.072 | -0.087 | -0.300** | -0.259* | -0.301* | -0.289* |
| FEV1% | 0.002 | -0.014 | -0.008 | -0.029 | -0.017 | -0.055 | -0.340** | -0.293* | -0.308* | -0.407*** |
| DLCO% | 0.343* | -0.183 | -0.101 | -0.095 | -0.321* | -0.290* | -0.375** | -0.290* | -0.286* | -0.461** |
| KCO% | -0.267* | -0.165 | -0.029 | -0.123 | -0.242 | -0.203 | -0.230 | -0.252* | -0.158 | -0.299* |
| PASP, mmHg | 0.351 | 0.367 | 0.250 | 0.393* | 0.376* | 0.320 | 0.626*** | 0.570** | 0.495* | 0.501* |

Level of statistical significance of Rho de Spearman: *p<0.05; **p<0.005; ***p<0.001

NLR, neutrophils to lymphocytes ratio; PLR, platelets to lymphocytes ratio; MLR, monocytes to lymphocytes ratio; PHR, platelets to hemoglobin ratio; SII, systemic inflammation immune index; PIV, pan-immune-inflammation values; CRP, C reactive protein; FCV, forced expiratory volume; FEV1, forced expiratory volume in the first second; DLO, diffusing capacity of the lung for carbon monoxide; KCO, Carbon monoxide transfer coefficient; PASP, pulmonary artery systolic pressure.

Supplementary Table 3.

|  | NLR | PLR | MLR | PHR* | SII | PIV | CRP at protocol | CRP to albumin ratio | CRP to prealbumin ratio | Average CRP |
| --- | --- | --- | --- | --- | --- | --- | --- | --- | --- | --- |
| Sex |  |  |  |  |  |  |  |  |  |  |
| Women, mean (SD) | 3.5 (4.5) | 174.5 (178.3) | 0.4 (0.6) | 195.5 (65.3) | 950.9 (1378.6) | 687.7 (1322.1) | 7.53 (8.2) | 1.98 (2.3) | 0.36 (0.4) | 6.5 (6.6) |
| Men, mean (SD) | 2.4 (1.1) | 184.1 (92.7) | 0.4 (0.1) | 189.1 (31.9) | 610.2 (284.0) | 360.6 (219.5) | 8.0 (4.6) | 2 (1.2) | 0.3 (0.1) | 7.7 (9.4) |
| *p-value* | 0.522 | 0.635 | 0.619 | 0.954 | 0.918 | 0.918 | 0.327 | 0.209 | 0.315 | 0.738 |
| Ethnicity |  |  |  |  |  |  |  |  |  |  |
| Caucasians, mean (SD) | 3.4 (4.4) | 174.4 (184.3) | 0.4 (0.6) | 192.1 (62.3) | 885.89 (1255.6) | 591.0 (1094.0) | 6.4 (7.1) | 1.6 (1.9) | 0.3 (0.3) | 5.5 (4.2) |
| Non-Caucasians, mean (SD) | 4.1 (4.8) | 177.5 (108.9) | 0.4 (0.3) | 216.0 (76.1) | 1283.3 (1928.4) | 1111.9 (2063.2) | 14.3 (11.6) | 4.0 (3.5) | 0.8 (0.7) | 14.4 (14.9) |
| *p-value* | 0.883 | 0.610 | 0.334 | 0.253 | 0.375 | 0.107 | **0.005** | **0.025** | **0.008** | **0.010** |
| Socioeconomic level |  |  |  |  |  |  |  |  |  |  |
| Low, mean (SD) | 3.92 (6.1) | 190.05 (248.1) | 0.5 (0.8) | 190.45 (70.6) | 1074.31 (181.2) | 820.72 (1721.0) | 8.49 (9.2) | 2.29 (2.6) | 0.41 (0.5) | 7.3 (8.4) |
| Medium/high, mean (SD) | 3.1 (2.0) | 158.8 (66.1) | 0.4 (0.2) | 197.2 (58.6) | 826.7 (803.2) | 529.6 (639.6) | 6.28 (6.6) | 1.61 (1.7) | 0.3 (0.3) | 5.9 (4.9) |
| *p-value* | 0.367 | 0.792 | 0.610 | 0.561 | 0.460 | 0.578 | 0.090 | 0.218 | 0.444 | 0.658 |
| Smoking status |  |  |  |  |  |  |  |  |  |  |
| Ever, mean (SD) | 4.18 (6.4) | 211.54 (272.9) | 0.6 (1.0) | 206.1 (65.2) | 1179.1 (1797.6) | 883.2 (1619.2) | 9.2 (9.8) | 2.3 (2.5) | 0.39 (0.4) | 7.4 (5.4) |
| Never, mean (SD) | 3.0 (2.5) | 153.0 (67.1) | 0.4 (0.2) | 188.8 (63.5) | 795.1 (998.4) | 560.3 (1062.3) | 6.6 (6.8) | 1.8 (2.1) | 0.3 (0.38) | 6.1 (7.3) |
| *p-value* | 0.528 | 0.457 | 0.292 | 0.236 | 0.101 | **0.033** | 0.305 | 0.304 | 0.845 | 0.079 |
| Anti-centromeres status |  |  |  |  |  |  |  |  |  |  |
| Positive, mean (SD) | 3.4 (5.3) | 177.41 (224.3) | 0.46 (0.8) | 181.52 (55.5) | 835.02 (1415.6) | 586.7 (1296.5) | 6.32 (6.3) | 1.62 (1.6) | 0.32 (0.3) | 5.3 (3.4) |
| Negative, mean (SD) | 3.5 (3.0) | 171.5 (78.4) | 0.4 (0.2) | 213.2 (71.1) | 1073.7 (1279.4) | 755.8 (1221.5) | 9.14 (9.8) | 2.48 (2.8) | 0.41 (0.5) | 8.2 (9.1) |
| *p-value* | 0.110 | 0.139 | 0.295 | **0.025** | **0.029** | **0.018** | 0.293 | 0.202 | 0.830 | 0.093 |
| Anti-Scl70 status |  |  |  |  |  |  |  |  |  |  |
| Positive, mean (SD) | 4.1 (4.0) | 186.02 (91.2) | 0.4 (0.2) | 235.3 (80.5) | 1360.8 (1714.5) | 1101.0 (1752.6) | 11.4 (11.6) | 3.2 (3.5) | 0.5 (0.6) | 10.9 (12.11) |
| Negative, mean (SD) | 3.29 (4.5) | 171.75 (193.1) | 0.4 (0.7) | 184.2 (54.7) | 821.6 (1228.3) | 460.0 (1119.0) | 6.48 (6.5) | 1.7 (1.7) | 0.3 (0.3) | 5.4 (3.4) |
| *p-value* | 0.359 | 0.127 | 0.284 | **0.006** | 0.075 | **0.048** | **0.013** | **0.018** | 0.469 | **0.035** |
| SSc-ILD |  |  |  |  |  |  |  |  |  |  |
| Yes, mean (SD) | 4.0 (3.5) | 169.8 (87.7) | 0.4 (0.2) | 222.2 (76.2) | 1353.87 (1547.9) | 1050.48 (1486.1) | 11.1 (11.1) | 3.1 (3.3) | 0.54 (0.5) | 10.5 (10.7) |
| No, mean (SD) | 3.3 (5.2) | 181.6 (87.7) | 0.5 (0.8) | 182.5 (54.5) | 810.6 (1407.5) | 548.7 (1284.1) | 6.28 (6.1) | 1.6 (1.5) | 0.29 (0.3) | 5.3 (3.5) |
| *p-value* | 0.024 | 0.634 | 0.150 | **0.012** | **0.012** | **0.002** | **0.030** | 0.074 | 0.210 | **0.004** |
| Respiratory damage |  |  |  |  |  |  |  |  |  |  |
| Yes, mean (SD) | 5.3 (7.3) | 240.5 (307.4) | 0.6 (1.0) | 221.7 (67.9) | 1545.9 (2135.2) | 1240.2 (2045.9) | 10.5 (11.0) | 2.83 (3.2) | 0.51 (0.6) | 9.8 (10.0) |
| No, mean (SD) | 2.7 (1.8) | 148.1 (60.4) | 0.3 (0.2) | 184.3 (60.0) | 676.8 (701.1) | 410.0 (556.2) | 6.34 (6.2) | 1.63 (1.7) | 0.29 (0.3) | 5.1 (3.8) |
| *p-value* | **0.004** | 0.084 | **0.017** | **0.016** | **<0.001** | **<0.001** | 0.134 | 0.128 | 0.331 | **0.002** |
| Heart disease |  |  |  |  |  |  |  |  |  |  |
| Yes, mean (SD) | 8.1 (12.8) | 385.2 (551.7) | 1.11 (1.9) | 217.3 (69.3) | 2232.2 (3316.2) | 1752.53 (2986.4) | 10.14 (13.1) | 2.75 (3.4) | 0.46 (0.7) | 8.0 (5.0) |
| No, mean (SD) | 3.0 (2.5) | 156.3 (69.9) | 0.4 (0.2) | 193.9 (64.1) | 824.9 (984.2) | 565.2 (953.73) | 7.3 (7.6) | 1.9 (2.2) | 0.3 (0.4) | 6.4 (6.9) |
| *p-value* | 0.284 | 0.142 | 0.315 | 0.360 | 0.102 | 0.106 | 0.944 | 0.694 | 0.534 | 0.142 |
| Cardiovascular damage |  |  |  |  |  |  |  |  |  |  |
| Yes, mean (SD) | 8.1 (11.2) | 333.8 (468.3) | 1.0 (1.6) | 245.0 (121.4) | 2424.9 (3214.6) | 2119.5 (3067.7) | 32.0 (9.9) | 8.7 (1.03) | 1.63 (0.39) | 33.22 (24.5) |
| No, mean (SD) | 2.8 (1.8) | 153.1 (61.1) | 0.3 (0.2) | 192.7 (64.6) | 9.2 (672.0) | 460.1 (542.9) | 7.0 (7.3) | 1.9 (2.1) | 0.3 (0.4) | 6.1 (4.5) |
| *p-value* | 0.071 | 0.257 | **0.003** | 0.303 | 0.061 | 0.014 | 0.017 | 0.137 | 0.073 | 0.0.78 |
| Glucocorticoids |  |  |  |  |  |  |  |  |  |  |
| Ever, mean (SD) | 4.4 (6.1) | 205.3 (242.0) | 0.6 (0.8) | 198.9 (72.5) | 1273.3 (1860.0) | 976.3 (1731.0) | 9.5 (10.2) | 2.6 (2.9) | 0.5 (0.5) | 8.4 (2.8) |
| Never, mean (SD) | 2.5 (0.9) | 145.1 (52.4) | 0.3 (0.1) | 191.8 (55.8) | 611.8 (278.1) | 351.4 (230.2) | 5.6 (4.7) | 1.41 (1.2) | 0.25 (0.2) | 4.7 (8.7) |
| *p-value* | 0.190 | 0.219 | **0.004** | 0.619 | 0.270 | **0.027** | 0.089 | 0.105 | 0.069 | **0.020** |
| Immunosuppressant |  |  |  |  |  |  |  |  |  |  |
| Any class |  |  |  |  |  |  |  |  |  |  |
| Ever, mean (SD) | 4.11 (5.36) | 194.35 (213.32) | 0.51 (0.72) | 192.3 (69.34) | 1131.31 (1643.76) | 840.56 (1529.96) | 8.26 (9.13) | 2.21 (2.57) | 0.4 (0.46) | 7.3 (7.92) |
| Never, mean (SD) | 2.25 (0.69) | 133.72 (44.1) | 0.28 (0.08) | 198.15 (52.72) | 572.8 (260.4) | 319.64 (197.86) | 5.42 (4.45) | 1.37 (1.06) | 0.26 (0.21) | 5 (3.01) |
| *p-value* | 0.059 | 0.198 | **0.002** | 0.642 | 0.209 | 0.138 | 0.390 | 0.939 | 0.668 | 0.393 |
| Non-Methotrexate |  |  |  |  |  |  |  |  |  |  |
| Ever, mean (SD) | 5.45 (7.57) | 233.37 (310.01) | 0.6 (10.6) | 212.0 (81.7) | 1627.1 (2283.12) | 1315.26 (2202.3) | 11.2 (12.1) | 3.1 (3.51) | 0.54 (0.6) | 10.13 (10.82) |
| Never, mean (SD) | 2.7 (1.6) | 152.0 (59.4) | 0.4 (0.2) | 189.2 (55.2) | 662.9 (507.3) | 430.8 (526.8) | 6.09 (5.2) | 1.57 (1.4) | 0.28 (0.2) | 5.1 (3.0) |
| *p-value* | **0.016** | 0.285 | 0.110 | 0.220 | **0.021** | **0.018** | 0.092 | 0.612 | 0.124 | **0.030** |
|  |  |  |  |  |  |  |  |  |  |  |

* Contrasts based on T-test for independent samples.

Supplementary Table 4. Eigenvalues and variance explained by components in principal component analysis (PCA) for Systemic Sclerosis following varimax rotation.

| Component | Initial eigenvalues | | |
| --- | --- | --- | --- |
|  | Eigenvalue | Variance (%) | Cumulative Variance (%) |
| 1 | 6.164 | 77.056 | 77.056 |
| 2 | 1.460 | 18.251 | 95.307 |
| 3 | 0.261 | 3.261 | 98.568 |
| 4 | 0.052 | 0.652 | 99.220 |
| 5 | 0.035 | 0.436 | 99.657 |
| 6 | 0.021 | 0.257 | 99.914 |
| 7 | 0.005 | 0.058 | 99.972 |
| 8 | 0.002 | 0.028 | 100.000 |

Extraction method: principal component analysis.

Supplementary T able 5. Patient characteristics stratified according to these 2 new groups

|  | Non-inflammatory Group  (N=72) | Inflammatory Group  (N=7) | *p*-value |
| --- | --- | --- | --- |
| Age, media (SD), yrs | 58.3 (11.2) | 60.9 (7.0) | 0.548 |
| Sex, Women, n (%) | 69 (95.8) | 7 (100) | 1.0 |
| Ethnicity, Caucasian, n (%) | 64 (88.9) | 4 (57.1) | 0.052 |
| Ever smoked, n (%) | 26 (36.1) | 3 (42.9) | 0.703 |
| Alcohol, n (%) | 4 (5.6) | 0 | 1.0 |
| Low socioeconomic level | 35 (48.6) | 5 (71.4) | 0.432 |
| Age-CCI, median (IQR) | 2.0 (1.0) | 4.0 (2.0) | **0.018** |
| SCORE, median (IQR) | 1.0 (2.0) | 2.0 (2.0) | 0.854 |
| Duration of SSc, median (IQR) | 9.5 (11.7) | 8.5 (20.7) | 0.710 |
| Disease duration ≤5 years, n (%) | 26 (36.1) | 2 (28.6) | 1.0 |
| Duration of CRP observation, median (IQR), yrs | 1.0 (3.0) | 8.0 (5.0) | 0.787 |
| Disease Classification |  |  |  |
| lcSSc, n (%) | 58 (80.6) | 6 (85.7) | 1.0 |
| dcSSc, n (%) | 13 (18.1) | 6 (85.7) | 1.0 |
| MRSS | 6.0 (8.0) | 12.0 (20.0) | 0.190 |
| Anemia, n (%) | 14 (19.4) | 2 (28.6) | 0.525 |
| Puffy fingers, n (%) | 28 (38.9) | 3 (42.9) | 1.0 |
| Sclerodactyly, n (%) | 56 (77.8) | 6 (85.7) | 1.0 |
| Digital tip ulcers, n (%) | 16 (222) | 3 (42.9) | 0.350 |
| Abnormal nail capillaries, n (%) | 63 (96.9) | 3 (75.0) | 0.166 |
| Dilated nail capillaries, n (%) |  |  |  |
| Low capillary density, n (%) |  |  |  |
| Arthralgia, n (%) | 47 (65.3) | 6 (85.7) | 0.416 |
| Arthritis, n (%) | 12 (16.7) | 2 (28.6) | 0.601 |
| Tendon friction rubs, n (%) | 4 (5.6) | 0 | 1.0 |
| Myalgia, n (%) | 4 (5.6) | 1 (14.3) | 0.379 |
| Calcinosis, n (%) | 24 (33.3) | 1 (14.3) | 0.422 |
| Persistent digestive symptoms, n (%) | 64 (88.9) | 6 (85.7) | 0.586 |
| Renal crisis, n (%) | 1 (1.4) | 1 (14.3) | 0.170 |
| Pericarditis, n (%) | 0 | 1 (14.3) | 0.089 |
| Dyspnea, n (%) | 21 (29.2) | 6 (85.7) | **0.006** |
| SSc-ILD, n (%) | 17 (27.0) | 6 (85.7) | **0.004** |
| NSIP, n (%) | 14 (22.2) | 4 (57.1) |  |
| Fibrotic NSIP, n (%) | 2 (3.2) | 2 (28.6) |  |
| UIP, n (%) | 1 (1.6) | 0 |  |
| HAP, n (%) | 5 (7.0) | 1 (14.3) | 0.442 |
| PSP, media (SD), mmHg | 31.6 (8.0) | 48.3 (10.4) | **0.003** |
| Respiratory Function Tests |  |  |  |
| FVC, media (SD), % | 84.0 (19.6) | 64.6 (25.0) | **0.018** |
| FEV1, media (SD), % | 88.3 (19.2) | 66.9 (23.5) | **0.008** |
| FEV1/FVC, media (SD), % | 104.9 (11.0) | 102.9 (25.8) | 0.739 |
| DLCO, media (SD), % | 67.7 (20.6) | 45.4 (13.4) | **0.021** |
| KCO, media (SD), % | 80.4 (18.0) | 63.5 (7.7) | 0.070 |
| FVC/DLCO, media (SD) | 1.3 (0.4) | 1.5 (0.2) | 0.239 |
| SCTC DI, median (IQR) | 3.0 (5.0) | 7.0 (13.0) | **0.014** |
| Weighted MSK & skin domain, median (IQR) | 0.0 (3.0) | 0.0 (0.3) | 0.600 |
| Weighted Vascular domain, median (IQR) | 0.0 (2.0) | 0.0 (2.0) | 0.569 |
| Weighted Gastrointestinal domain, median (IQR) | 0.0 (1.0) | 1.0 (1.0) | **0.033** |
| Weighted Respiratory domain, median (IQR) | 0.0 (2.0) | 4.0 (6.0) | **0.005** |
| Weighted CV domain, median (IQR) | 0.0 (0.0) | 0.0 (3.0) | **0.007** |
| Weighted Renal domain, median (IQR) | 0.0 (0.0) | 0.0 (0.0) | **0.039** |
| SCTC >= 13, n (%) | 2 (2.8) | 3 (42.9) | **0.004** |
| ANA, n (%) |  |  |  |
| Anti-centromere proteins, n (%) | 45 (62.5) | 2 (28.6) | 0.113 |
| Anti-Scl70+, n (%) | 11 (15.3) | 3 (42.9) | 0.102 |
| Anti-PM/Scl, n (%) | 4 (6.1) | 0 | 1.0 |
| Anti-Ku, n (%) | 2 (3.0) | 0 | 1.0 |
| Anti-U1-RNP, n (%) | 4 (6.0) | 0 | 1.0 |
| Treatment |  |  |  |
| Immunosuppressors, n (%) | 49 (68.1) | 6 (85.7) | 0.669 |
| Non-methotrexate IS, n (%) | 17 (23.9) | 5 (71.4) | **0.017** |
| Methotrexate, n (%) | 34 (47.2) | 4 (571) | 0.705 |
| Hydroxychloroquine, n (%) | 14 (19.4) | 0 | 0.342 |
| Mycophenolate mofetil, n (%) | 16 (22.2) | 5 (71.4) | **0.013** |
| Cyclophosphamide, n (%) | 1 (1.4) | 3 (42.9) | **0.002** |
| Antifibrotic, n (%) | 2 (25.0) | 1 (100) | **0.333** |
| Rituximab, n (%) | 2 (2.8) | 1 (14.3) | 0.249 |
| Tocilizumab, n (%) | 2 (2.8) | 0 | 1.0 |
| Prednisone, n (%) | 33 (45.8) | 6 (85.7) | 0.057 |
| Calcium Channel Blockers, n (%) | 54 (75.0) | 6 (85.7) | 1.0 |
| PDE5 inhibitors, n (%) | 12 (16.7) | 2 (28.6) | 0.601 |
| Endothelin receptor antagonists, n (%) | 10 (13.9) | 1 (14.3) | 1.0 |
| ACE inhibitors, n (%) | 9 (12.7) | 1 (14.3) | 1.0 |
| Neutrophils, median (IQR), 10^9^/mL | 4.3 (1.6) | 10.4 (4.4) | **0.011** |
| Lymphocytes, median (IQR), 10^9^/mL | 1.6 (0.8) | 1.5 (1.0) | 0.474 |
| Monocytes, median (IQR), 10^9^/mL | 0.5 (0.2) | 0.9 (0.3) | **0.002** |
| Platelets, media (SD), 10^9^/mL | 238.2 (68.4) | 351.0 (80.4) | **<0.001** |
| Neutrophils to Lymphocytes ratio, median (IQR) | 2.3 (1.4) | 7.4 (14.3) | **0.002** |
| Monocytes to Lymphocytes ratio, median (IQR) | 0.3 (0.1) | 0.5 (0.9) | **0.045** |
| Platelets to Lymphocytes radio, median (IQR) | 140.0 (65.0) | 246.7 (239.1) | **0.007** |
| Platelets to Hemoglobin ratio, media (SD) | 185.8 (56.0) | 272.4 (92.6) | **0.048** |
| Systemic inflammation immune index, median (IQR) | 540.7 (383.8) | 2412.2 (5532.8) | **<0.001** |
| Pan-immune inflammation values, median (IQR) | 293.9 (338.7) | 1834.1 (6067.9) | **<0.001** |
| CRP, mg/L, median (IQR) | 4.0 (0.0) | 28.0 (14.0) | **<0.001** |
| CRP/Albumin, median (IQR) | 1.0 (0.1) | 8.0 (2.7) | **<0.001** |
| CRP/Pre-albumin, median (IQR) | 0.2 (0.1) | 1.3 (0.8) | **<0.001** |
| Average CRP, mg/L, median (IQR) | 4.0 (3.6) | 15.9 (14.3) | **<0.001** |
| Persistent inflammatory phenotype, n 8%) | 4 (5.6) | 4 (57.1) | **0.001** |
